# Expectoration of *Cryptosporidium* Parasites in Sputum of Human Immunodeficiency Virus–Positive and –Negative Adults

**DOI:** 10.4269/ajtmh.17-0741

**Published:** 2018-02-05

**Authors:** Siobhan M. Mor, Luke R. Ascolillo, Ritah Nakato, Grace Ndeezi, James K. Tumwine, Alphonse Okwera, Jerlyn K. Sponseller, Saul Tzipori, Jeffrey K. Griffiths

**Affiliations:** 1 University of Sydney School of Veterinary Science, Sydney, Australia;; 2 Tufts University School of Medicine, Boston, Massachusetts;; 3 Makerere University College of Health Sciences, Kampala, Uganda;; 4 Tufts University Cummings School of Veterinary Medicine, Grafton, Massachusetts

## Abstract

Respiratory cryptosporidiosis is thought to be a rare, end-stage complication of HIV. Few studies have systematically examined the frequency of such infection in adults. Sputum specimens submitted for tuberculosis (TB) testing at Mulago Hospital, Uganda, were anonymously retested for *Cryptosporidium* using real-time polymerase chain reaction (PCR). Visual confirmation using immunofluorescence confocal microscopy was performed for a subset of PCR-positive samples. Of 824 sputum samples tested, 24 (2.9%) were *Cryptosporidium* positive. Prevalence in sputum ranged between 0% and 10% in each month of the study and exceeded TB prevalence in some months. In this referral population, respiratory *Cryptosporidium* prevalence was lower in people with HIV (1.3% versus 4.4% without HIV, *P* = 0.028) and higher in those with TB (6.8% versus 2.6% without TB, *P* = 0.086). The weak association between respiratory *Cryptosporidium* infection and TB persisted after controlling for HIV (odds ratio = 3.2, 95% confidence interval: 0.9, 11.8; *P* = 0.080). This is the first study to document adult respiratory tract cryptosporidiosis in a referral population with presumed TB. These findings 1) confirm that *Cryptosporidium* respiratory infection occurs in HIV-negative and -positive adults; 2) suggest there is potential for *Cryptosporidium* to be disseminated or transmitted by coughing or expectoration; and 3) identify possible synergy between *Cryptosporidium* and TB in the respiratory tract.

## INTRODUCTION

Cryptosporidiosis is a common, emerging disease, unrecognized before the AIDS pandemic, and historically underappreciated as a cause of childhood diarrhea and death. Recently, the Global Enteric Multicenter Study revealed the parasite to be the second leading cause of moderate-to-severe diarrhea in infants in five of seven sites across Africa and Asia,^1^ with an estimated 2.9 million cases in sub-Saharan Africa alone.^2^ Indeed, in Uganda, we have found that 25% of children presenting with diarrhea to the national referral hospital have *Cryptosporidium* sp. in their stool and the 2-week case fatality rate in such children is 13%.^3^ Others have documented growth retardation, malnutrition, and death in children elsewhere in Africa as a result of *Cryptosporidium* infection.^4,5^ No effective therapy for cryptosporidiosis exists; nitazoxanide shortens diarrhea duration in normal hosts by 1–4 days, and is ineffective in people with HIV/AIDS.^6–9^ Thus, population-wide protection against this pathogen is primarily through the prevention of transmission, which is understood to occur via ingestion of parasites through direct contact with infected people or animals, or indirectly via ingestion of contaminated food and/or water.

Until recently, respiratory tract infection with *Cryptosporidium* spp. was thought to be a rare complication of intestinal disease in people with HIV or other immunosuppressive conditions.^10^ Limited case reports and case series documented respiratory tract infection through visualization of oocysts in sputum and bronchiolar lavage samples,^11–16^ as well as biopsy specimens.^11,15,17,18^ We recently found that respiratory tract involvement in fact occurs frequently in children with enteric cryptosporidiosis in Uganda, with as many as 35% of children with cough and intestinal infection having parasite DNA in respiratory secretions.^19^ Contrary to current understanding, most of these children were HIV negative.

Adults are less likely to exhibit the symptoms of enteric cryptosporidiosis unless they are immunocompromised. Aside from the abovementioned case reports in people with HIV/AIDS, no studies have systematically investigated how common respiratory tract infection is in adults. In this study, we retested sputum samples obtained for tuberculosis (TB) testing from adults in Uganda. This approach afforded an efficient, low-cost means to investigate how frequently respiratory infection occurs in an adult population enriched with HIV-positive individuals.

## MATERIALS AND METHODS

### Study population and specimen collection.

De-identified, expectorated sputum specimens that were otherwise-to-be-discarded were obtained from the TB reference laboratory at Mulago Hospital, Kampala, Uganda. Samples received by this laboratory come from in- and out-patients attending the hospital or, more frequently, satellite clinics. Per the algorithm used in the unit, TB testing for known HIV-positive cases is performed using GeneXpert, whereas HIV-negative cases are screened for TB using fluorescent smear microscopy (except where the clinician specifically requests GeneXpert). Satellite centers refer samples mainly for GeneXpert; thus, a substantial proportion of specimens are from known HIV-positive people who are already initiated on antiretroviral treatment and who are presumed to have TB. Only first-time submissions to the TB reference laboratory from adults aged ≥ 18 years were included in this study, so that each specimen represents a unique individual.

In addition to sputum specimens, basic information on sex, age (in years), and HIV status (if known) of the anonymous donor as well as outcome of TB testing was made available for analysis. Given that anonymous donors presented to Mulago Hospital for TB testing, no data were available on diarrheal illness at the time of the visit nor was a stool sample collected. Sputum samples analyzed in this study were obtained between April 2014 and November 2015. No samples were obtained between February 10 and April 12, 2015 because of changes in the study protocol that were being implemented at the time.

### Laboratory analysis.

Up to 150 μL of sputum was spotted onto FTA cards (Whatman, Inc., Clifton, NJ) and air-dried at room temperature. FTA cards were shipped to Tufts University, Boston, MA, where the DNA was eluted according to the manufacturer recommendations. Eluates were initially screened using a SYBR-based, nested polymerase chain reaction (PCR) assay targeting the 18S rRNA gene.^20^ For the primary PCR, reactions were performed in a volume of 50 μL consisting of 25 μL ReadyMix Taq PCR Reaction Mix (Sigma-Aldrich, St. Louis, MO), 5 μL each primer (0.5 μM final concentration), 7 μL of extracted DNA, and 8 μL of PCR grade water. Thermocycling conditions consisted of a denaturation step of 94°C for 1 minute followed by 25 cycles of 55°C for 2 minutes and 72°C for 3 minutes. For the secondary PCR, reactions were performed in a volume of 25 μL consisting of 12.5 μL iTaq™ Universal SYBR^®^ Green Supermix (Bio-Rad Laboratories, Hercules, CA), 2.75 μL each primer (0.25 μM final concentration), and 7 μL of extracted DNA. Thermocycling conditions for the second round of PCR consisted of 95°C for 3 minutes followed by 40 cycles of 95°C for 15 seconds and then 63°C for 50 seconds.

Samples that were given a positive determination (cycle threshold value of ≤ 33) by the nested PCR were subjected to a further round of real-time PCR using species-specific probes designed to distinguish *Cryptosporidium hominis* (small subunit rRNA gene), *Cryptosporidium parvum* (SSU rRNA gene), and *Cryptosporidium meleagridis* (60-kDa glycoprotein 60). Primer and probe sets for *C. parvum* and *C. hominis* were those previously described by Hadfield et al.^21^ For the purposes of multiplexing, *C. parvum* and *C. hominis* were labeled with FAM and HEX, respectively (Applied Biosystems, Foster City, CA). The *C. meleagridis* primer and probe set was designed using software from Integrated DNA Technologies (IDT, Coralville, IA) using GenBank sequence KF733822 and labeled with Texas Red (IDT). Primers and probes were reconstituted to 100 μM concentrations as necessary. All primers were added for a final concentration of 900 nM per reaction, and the probes were at a concentration of 300 nM per reaction. A master mix containing iQ Multiplex Powermix (BioRad) (12.5 μL per reaction) and primers, probes, and reagent water was made in batches. Reactions were performed in a volume of 25 μL containing 18 μL of master mix and 7 μL of extracted DNA. Thermocycling conditions consisted of a 3-minute denaturation step at 95°C followed by 45 cycles of 95°C for 15 seconds and 60°C for 50 seconds. A full list of primer/probe sequences used in this study is presented in Supplemental Table 1. For quality control purposes, a subset of PCR-positive samples were subjected to visual confirmation using immunofluorescence and confocal microscopy of sputum smears prepared in Uganda. Oocysts were visualized using a commercial kit containing a fluorescein-labeled mouse monoclonal antibody (Crypt-a-Glo; Waterborne Inc., New Orleans, LA). Of the first ∼300 samples tested using PCR, all but one positive was confirmed using immunofluorescence.

### Sample size and statistical analysis.

The primary outcome of interest was the presence or absence of *Cryptosporidium* spp. in sputum from adults undergoing TB testing. Using the binomial formula, we calculated that a sample size of 4,000 would allow us to address, with 98% confidence, the primary hypothesis that the prevalence in sputum was not greater than 0.1%. Secondary outcomes of interest included species of *Cryptosporidium* in sputum and host factors associated with the primary outcome (age, gender, HIV status, and TB test result). The study was terminated before achieving this sample size target because it became clear that the original hypothesis—that prevalence in sputum was not greater than 0.1%—could be refuted and secondary outcomes could be addressed within the constraints of the anonymous retesting study design.

To assess temporal patterns, the prevalence of respiratory *Cryptosporidium* infection and TB were reported separately by month. Host factors associated with respiratory *Cryptosporidium* status were analyzed using χ^2^ analysis (Fisher exact test). A logistic regression model was fitted to the data to explain predicted odds of having *Cryptosporidium*-positive sputum. Host factors with a *P* value < 0.1 were included in subsequent multivariate analysis. Analysis was performed in IBM SPSS Statistics (Version 21; IBM Corp., Armonk, NY). This research was reviewed and approved by the institutional review boards of the Tufts Medical Center/Tufts University Health Sciences (protocol 10699) and the Makerere University College of Health Sciences (protocol 2013-001).

## RESULTS

Between April 2014 and November 2015, 824 sputum samples were obtained from anonymous, adult donors. Table 1 shows the demographic and clinical characteristics of donors. Median age of donors was 30 years (range: 18–92). Prevalence of TB and HIV (if known) among the donors was 7.2% and 36.7%, respectively. TB was not associated with HIV in this referral population (7.7% in HIV-positive donors versus 6.4% in HIV-negative donors, *P* = 0.632).

**Table 1:** Demographic and clinical characteristics of 824 anonymous sputum donors

Characteristic		No. (%)
Age quartile, years	18–25	227 (27.5)
	26–30	206 (25.0)
	31–39	179 (21.7)
	≥ 40	212 (25.7)
Sex	Female	333 (40.4)
	Male	490 (59.5)
	Unknown	1 (0.1)
Tuberculosis	Positive	59 (7.2)
	Negative	765 (92.8)
HIV	Positive	302 (36.7)
	Negative	298 (36.2)
	Unknown	224 (27.2)

**Figure 1.**
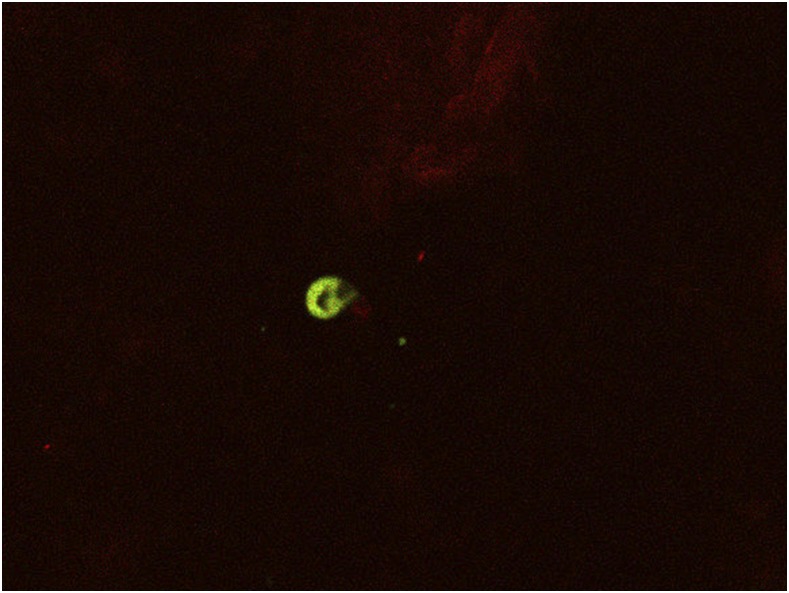
Immunofluroscence confocal microscopy of *Cryptosporidium*-positive sputum smear.

**Figure 2.**
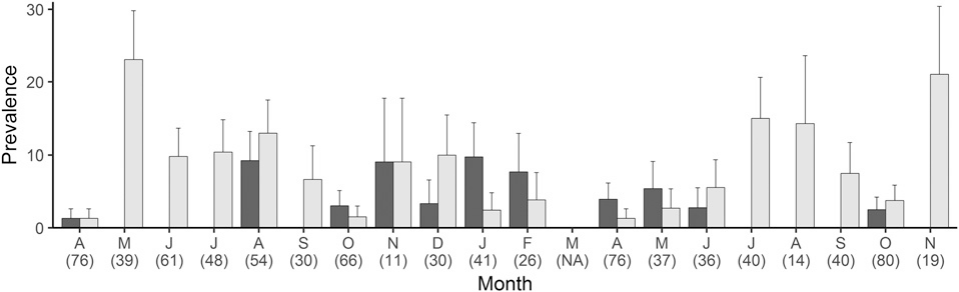
Prevalence of respiratory tract cryptosporidiosis (dark gray) and tuberculosis (light gray), by month, in anonymous sputum donors in Uganda (*N* = 824). Specimens were collected between April 2014 and November 2015. Number of specimens tested in each month is shown in parentheses. No specimens were obtained for the study in March 2015. Error bars = standard error of individual proportions.

Of 824 sputum specimens tested with PCR, 24 were positive for *Cryptosporidium* spp. (2.9%; 95% confidence interval [CI]: 1.7, 4.1%). Of these, 15 (62.5%) were *C. hominis* whereas 9 (37.5%) were *C. parvum*. Parasites were observed in several specimens that tested positive by PCR (Figure 1). Figure 2 shows the temporal pattern of *Cryptosporidium*-positive and TB-positive sputum specimens obtained during the study. Respiratory *Cryptosporidium* prevalence varied between 0% and 10% in each month of the study and exceeded TB prevalence in some months.

Table 2 shows the demographic and clinical characteristics of donors by *Cryptosporidium* status. Age and sex were not significantly associated with being sputum positive for *Cryptosporidium* in univariate analysis. Respiratory *Cryptosporidium* prevalence was lower in people with HIV (1.3% versus 4.4% without HIV; *P* = 0.028) and higher in donors with TB (6.8% versus 2.6% without TB, *P* = 0.086). These findings persisted in multivariate analysis. Specifically, donors with HIV and TB, respectively, were 0.3 (95% CI: 0.1, 0.9; *P* = 0.031) and 3.2 (95% CI: 0.9, 11.8; *P* = 0.08) times as likely to have *Cryptosporidium*-positive sputum. An interaction term between HIV and TB was nonsignificant.

**Table 2:** Sputum *Cryptosporidium* status of 824 anonymous sputum donors, by demographic and clinical characteristics

Characteristic		No. (%) positive	Odds ratio (95% confidence interval)	*P* value
Age quartile, years	18–25	6 (2.6)	1.9 (0.5, 7.7)	0.506
	26–30	8 (3.9)	2.8 (0.7, 10.7)	0.136
	31–39	7 (3.9)	2.8 (0.7, 11.1)	0.197
	≥ 40	3 (1.4)	1	–
Sex	Female	6 (1.8)	0.5 (0.2, 1.3)	0.197
	Male	17 (3.5)	1	–
	Unknown	1 (100.0)	–	–
Tuberculosis	Positive	4 (6.8)	2.7 (0.9, 8.2)	0.086
	Negative	20 (2.6)	1	–
HIV	Positive	4 (1.3)	0.3 (0.1, 0.9)	0.028
	Negative	13 (4.4)	1	–
	Unknown	7 (3.1)	–	–

## DISCUSSION

In our previous work, we showed that respiratory involvement occurs frequently in children with intestinal cryptosporidiosis in Uganda.^19^ Although there have been several historical case reports/series of respiratory *Cryptosporidium* infection in adults,^10^ no studies have systematically investigated the occurrence of respiratory cryptosporidiosis in adults in Africa or elsewhere. In the present study, we found that around 3% of adults presenting for TB testing in Uganda—10% in some months—had *Cryptosporidium* spp. in their sputum. The intestinal status of these anonymous, adult sputum donors was not known and therefore we are unable to comment on the frequency of respiratory involvement in adults with intestinal cryptosporidiosis. Nevertheless, the finding that a proportion of adults with presumptive TB are expectorating parasites in sputum was unanticipated. This finding is supported by the use of multiple diagnostic approaches, which included screening with a SYBR-based nested PCR protocol followed by confirmation using a probe-based assay, as well as immunofluorescence microscopy on a small number of samples.

Most of the studies on *Cryptosporidium* epidemiology and transmission have focused on diarrheal illness and have not attempted to ascertain respiratory symptoms or outcomes. Detection of *Cryptosporidium* in expectorated sputum of symptomatic adults suggests the potential for infected people to contaminate the environment via respiratory droplets. For an organism with a low infectious dose (10–30 oocysts^22^), we suggest this may prove an important strategy for parasite survival and persistence in a population. Furthermore, if respiratory transmission is confirmed, respiratory precautions, such as using masks, covering the mouth/nose when coughing, environmental surface cleaning, and patient isolation, may be advisable when managing patients infected with *Cryptosporidium*. In developed countries, outbreaks of cryptosporidiosis have been known to occur in hospitals, day-care centers, nurseries, and other congregate settings where such respiratory precautions may prove important.

Similar to our pediatric study,^19^ many of the adults with respiratory *Cryptosporidium* infection were HIV negative. Historically (i.e., before highly active antiretroviral therapy [HAART]), respiratory tract cryptosporidiosis was reported as a rare, late-stage complication of intestinal *Cryptosporidium* infection in people with AIDS. The lack of positive association between respiratory *Cryptosporidium* infection and HIV observed in this study is most likely due to referral bias. Indeed, there was no association between HIV and TB in this study population. We suspect this is because many patients are referred from specialized treatment centers, where most if not all will be on antiviral treatment, receiving nutritional support and counseling on hygiene behaviors to reduce opportunistic infections. Furthermore, some patients may be on anti-TB prophylaxis or taking azithromycin or similar drugs, either intermittently or on a weekly basis. Any of these would decrease the likelihood of being sputum positive for TB in persons with HIV, by improving immunity or having a direct anti-TB effect. Assuming immunological factors are important for respiratory tract cryptosporidiosis, it is conceivable that persons with treated HIV may have a similar frequency of respiratory tract involvement as people without HIV in areas where cryptosporidiosis is prevalent. We speculate that the lack of nutritional support and hygiene counseling in people without HIV may actually render them more susceptible to respiratory tract cryptosporidiosis than people with well-managed HIV. Regardless, our data confirm that—contrary to conventional understanding—HIV is not a necessary condition for respiratory tract cryptosporidiosis. Further studies are needed to improve the knowledge of the epidemiology and clinical significance of respiratory tract cryptosporidiosis in the post-HAART era.

We found a positive albeit nonsignificant (*P* = 0.08) association between respiratory tract cryptosporidiosis and TB in this study, which has not to our knowledge been previously reported. Given overlap in risk factors for *active* TB and cryptosporidiosis (e.g., HIV, malnutrition, or other immune deficient states), it is possible that this association is confounded by these or other factors. Again, an association with active TB would support the view that respiratory tract cryptosporidiosis is related to immunocompromise. Nonetheless, coinfection in the respiratory tract raises the possibility that local factors may alter the hosts propensity for infection at this site. Given constraints on the data imposed by the anonymous study design, we were not able to explore these possibilities in detail.

In this study, around 60% of the positive sputum specimens tested were *C. hominis*, whereas the remaining specimens were *C. parvum*. This differs slightly to our previous pediatric studies in Uganda, where around 75% of intestinal cases were caused by *C. hominis*, 20–25% by *C. parvum*, and the remainder caused by a mixture of these species or *C. meleagridis*.^3,23^ Other studies in Africa report a similar preponderance of *C. hominis* in diarrheal cases in children,^24^ leading to the conclusion that transmission is principally due to human-to-human spread (rather than zoonotic) in this environment. It is possible that this conclusion is biased by the tendency of studies to focus solely on children. Adults may experience more diverse exposures including, for instance, occupational exposure to animals. We do not believe that the higher prevalence of *C. parvum* reported here reflects an increased propensity of this species to infect the respiratory tract because *C. hominis* was the dominant species found in children with respiratory involvement in our previous research.^19^ Further studies in adults are needed to confirm or refute these hypotheses.

We focused on patients presenting to a TB testing center as this provided an efficient means to access large numbers of sputum specimens to test the primary hypothesis that the prevalence of *Cryptosporidium* in sputa was not greater than 0.1%. In addition, this population is enriched with people with HIV infection and per conventional understanding, they were anticipated to have higher rates of respiratory infection, thus potentially serving as “super-spreaders” if indeed droplet or aerosol transmission does occur. As noted previously, because of the referral patterns at this clinic, the findings presented here cannot be generalized to the general population or population with suspected TB. Given that sputa were expectorated, it is possible that the presence of parasites was due to contamination with saliva. Saliva was not tested in the present study because of the anonymous design. However, we note that none of the 17 children with confirmed respiratory cryptosporidiosis in our previous study had parasites in the saliva.^19^ Furthermore, of 103 saliva samples tested in that study, only two were positive (versus 17/48 sputum samples). On the basis of these findings, we believe it is unlikely that the results presented here are due to saliva contamination.

## CONCLUSIONS

This study is the first study to systematically investigate respiratory cryptosporidiosis in adults with suspected TB. Parasites were detected in expectorated sputum using PCR and immunofluorescence in both HIV-positive and -negative patients. A positive albeit insignificant association with TB was documented.

## Supplementary Material

Supplemental Figure.
