# Supplementary material for: Expectoration of Cryptosporidium Parasites in Sputum of Human Immunodeficiency Virus–Positive and –Negative Adults
Source: Am J Trop Med Hyg. 2018 Feb 5;98(4):1086–90. doi: 10.4269/ajtmh.17-0741 (PMC5928827; doi:10.4269/ajtmh.17-0741)
Supplement: Supplementary file 1 [file tpmd170741.SD1.pdf]

Supplemental Table 1

Polymerase chain reaction (PCR) primers used in this study

Target locus	Primer/probe	Sequence	Refs.
SYBR-based nested PCR			
Cryptosporidium SSU rRNA (primary amplification)	Forward	TTCTAGAGCTAATACATGCG	20
	Reverse	CCCATTTCCTTCGAAACAGGA	
Cryptosporidium SSU rRNA (secondary amplification)	Forward	GGAAGGGTTGTATTTATTAGATAAAG	
	Reverse	AAGGAGTAAGGAACAACCTCCA	
Probe-based assay			
Cryptosporidium parvum SSU rRNA	Forward	TCCTTGAAATGAATATTTGTGACTCG	21
	Reverse	TTAATGTGGTAGTTGCGGTTGAAC	
	Probe	FAM-TATCTCTTCGTAGCGGCGTA-BHQ1	
Cryptosporidium hominis SSU rRNA	Forward	TCCTTGAAATGAATATTTGTGACTCG	21
	Reverse	AAATGTGGTAGTTGCGGTTGAAA	
	Probe	VIC-CTTACTTCGTGGCGGCGT-BHQ1	
Cryptosporidium meleagridis 60 kDa glycoprotein 60	Forward	GAGCTCAGCACTCTCTCTACTA	Present study
	Reverse	GCGTCTGTGAGTGATCTTCTT	
	Probe	TEX615-ATGACACTGCGTCTGAGGAAAGCT-3IAbRQSp	

SSU = small subunit.
